# Longitudinal Analysis of Cancer Risk in Children and Adults With Germline *PTEN* Variants

**DOI:** 10.1001/jamanetworkopen.2023.9705

**Published:** 2023-04-24

**Authors:** Lamis Yehia, Gilman Plitt, Ann M. Tushar, Julia Joo, Carol A. Burke, Steven C. Campbell, Katherine Heiden, Judy Jin, Carole Macaron, Chad M. Michener, Holly J. Pederson, Kadakkal Radhakrishnan, Joyce Shin, Joan Tamburro, Sujata Patil, Charis Eng

**Affiliations:** 1 Genomic Medicine Institute, Lerner Research Institute, Cleveland Clinic, Cleveland, Ohio; 2 Department of General Surgery, Digestive Disease and Surgery Institute, Cleveland Clinic, Cleveland, Ohio; 3 Cleveland Clinic Lerner College of Medicine of Case Western Reserve University School of Medicine, Cleveland, Ohio; 4 Department of Gastroenterology, Hepatology and Nutrition, Digestive Disease and Surgery Institute, Cleveland Clinic, Cleveland, Ohio; 5 Sanford R. Weiss MD Center for Hereditary Colorectal Neoplasia, Digestive Disease and Surgery Institute, Cleveland Clinic, Cleveland, Ohio; 6 Department of Colorectal Surgery, Digestive Disease and Surgery Institute, Cleveland Clinic, Cleveland, Ohio; 7 *PTEN* Multidisciplinary Clinic and Center of Excellence, Cleveland Clinic, Cleveland, Ohio; 8 Department of Urology, Cleveland Clinic, Cleveland, Ohio; 9 Center for Endocrine Surgery, Cleveland Clinic, Cleveland, Ohio; 10 Division of Gynecologic Oncology, Obstetrics, Gynecology and Women's Health Institute, Cleveland Clinic, Cleveland, Ohio; 11 Department of Pediatric Gastroenterology, Hepatology, and Nutrition, Cleveland Clinic, Cleveland, Ohio; 12 Department of Dermatology, Cleveland Clinic, Cleveland, Ohio; 13 Department of Quantitative Health Sciences, Lerner Research Institute, Cleveland Clinic, Cleveland, Ohio; 14 Center for Personalized Genetic Healthcare, Community Care, Cleveland Clinic, Cleveland, Ohio; 15 Taussig Cancer Institute, Cleveland Clinic, Cleveland, Ohio; 16 Department of Genetics and Genome Sciences, Case Western Reserve University School of Medicine, Cleveland, Ohio; 17 Germline High Risk Cancer Focus Group, Case Comprehensive Cancer Center, Case Western Reserve University, Cleveland, Ohio

## Abstract

**Importance:**

Identifying hereditary cancer predisposition facilitates high-risk organ-specific cancer surveillance and prevention. In *PTEN* hamartoma tumor syndrome (PHTS), longitudinal studies remain lacking, and there are insufficient data on cancers in children and young adults, as well as individuals with neurodevelopmental disorders (NDD).

**Objective:**

To evaluate lifetime cancer risks, including second malignant neoplasms (SMN), among patients with PHTS.

**Design, Setting, and Participants:**

Prospective longitudinal cohort study (September 1, 2005, through January 6, 2022). General population risks from the Surveillance, Epidemiology, and End Results database. Patients with PHTS, molecularly defined as carrying germline *PTEN* variants, were accrued from community and academic medical centers throughout North America, South America, Europe, Australia, and Asia. Data were analyzed from July 2022 to February 2023.

**Exposures:**

Review of physical and electronic medical records, and follow-up through clinical visits or telephone interviews.

**Main Outcomes and Measures:**

Lifetime cancer risks in PHTS relative to the general population.

**Results:**

A total of 7302 patients were prospectively accrued, 701 of whom had germline *PTEN* variants (median [IQR] age at consent, 38 [12-52] years; 413 female patients [59%]). Longitudinal follow-up data could be obtained for 260 patients (37%), with a median (IQR) follow-up of 4 (2-8) years. Of the 701 patients, 341 (49%) received at least 1 cancer diagnosis, with 144 (42%) of those having SMN. The study found significantly elevated lifetime risks for breast (91%), endometrial (48%), thyroid (33%), kidney (30%), and colorectal cancers (17%), as well as melanoma (5%). Cancer diagnoses were also observed in children and young adults with PHTS (15%) and in patients with PHTS with neurodevelopmental disorders (11%). Elevated risks (*P* < .001) of thyroid (age-adjusted standardized incidence ratios [SIR], 32.1; 95% CI, 26.0-39.0), kidney (SIR, 26.5; 95% CI, 18.8-36.3), endometrial (SIR, 26.0; 95% CI, 19.5-34.1), breast (SIR, 20.3; 95% CI, 17.3-23.7), and colorectal (SIR, 7.9; 95% CI, 5.2-11.7) cancers, and melanoma (SIR, 6.3; 95% CI, 3.5-10.5) were observed. Of the 341 patients with PHTS with cancer, 51 (15%) had 1 or more cancers diagnosed at age 29 years or younger, and 16 (31.4%) of those developed SMN at final follow-up. Twenty-three patients with PHTS with NDD and cancer were identified, with 5 (22%) having developed SMN at final follow-up. Individuals with PHTS and NDD showed higher lifetime cancer risks compared with individuals with PHTS but without NDD (hazard ratio, 2.7; 95% CI, 1.7-4.2; *P* < .001).

**Conclusions and Relevance:**

This cohort study found consistently elevated lifetime cancer risks in PHTS. Organ-specific surveillance should continue in patients with PHTS. Additional study is required to ascertain elevated cancer risks in patients with PHTS with NDD.

## Key Points

**Question:**

What are the lifetime cancer risks in children and adults with germline *PTEN* variants?

**Findings:**

In this cohort study of 701 patients with germline *PTEN* variants (*PTEN* hamartoma tumor syndrome, PHTS), approximately half had at least 1 cancer diagnosis, with significantly elevated lifetime risks for breast (91%), endometrial (48%), thyroid (33%), kidney (30%), and colorectal cancers (17%), as well as melanoma (5%). Cancer diagnoses were also observed in children and young adults with PHTS (15%) and in patients with PHTS with neurodevelopmental disorders (11%).

**Meaning:**

These findings suggest that, according to the consistently demonstrated elevated lifetime cancer risks, organ-specific surveillance should continue in patients with germline *PTEN* variants.

## Teaser

This cohort study evaluates the cancer spectrum and risks in children and adults with germline *PTEN* variants.

## Introduction

*PTEN* hamartoma tumor syndrome (PHTS) encompasses individuals with pathogenic germline *PTEN* variants regardless of clinical diagnosis.^1,2^ PHTS includes 4 clinically distinct allelic overgrowth syndromes, namely Cowden syndrome and Bannayan-Riley-Ruvalcaba syndrome, as well as *PTEN-*related Proteus and Proteus-like syndromes.^1^ *PTEN* is a tumor suppressor gene encoding phosphatase and tensin homologue (PTEN), a ubiquitously expressed phosphatase that counteracts the growth-promoting phosphoinositide 3-kinase/protein kinase B/mammalian target of rapamycin signaling cascade, and has other protean functions within various cellular compartments.^3,4^ Studies in adult patients with PHTS indicate that germline *PTEN* variants are associated with increased lifetime risks of breast, thyroid, endometrial, kidney, and colon cancers, as well as melanoma.^5,6,7,8^ Patients with PHTS also have notably elevated lifetime risks of second primary malignant neoplasms (SMN) compared with the general population.^9^ Intriguingly, up to 23% of patients with PHTS have neurodevelopmental disorders (NDD), including autism spectrum disorder (ASD), developmental delay, and/or intellectual disability, with *PTEN* being one of the most common predisposition genes for ASD.^2,10^ Therefore, in addition to being a classical cancer-related gene, germline *PTEN* variants are one of the most common monogenic causes of NDD.^11,12,13^

The ability to recognize and molecularly diagnose PHTS facilitates the implementation of high-risk organ-specific cancer surveillance strategies that result in early detection and prevention.^1^ The goal of enhanced cancer surveillance is to detect any malignant neoplasms at the earliest, most treatable stages. Despite multiple studies investigating cancer risks in PHTS, longitudinal studies remain lacking. Additionally, there are insufficient data on the frequency and spectrum of cancers observed in children and young adults with PHTS, as studies have mainly focused on adults. Importantly, another knowledge gap in the *PTEN* field is whether individuals with PHTS and NDD have similar lifetime cancer risks compared with neurotypical individuals with PHTS. In this study, we report results from the longitudinal follow-up of a prospective international series of pediatric and adult patients with PHTS. We investigate age-related cancer risks and modifying *PTEN* genotype-associated variables to inform prospective cancer risk assessment and surveillance recommendations.

## Methods

### Research Participants

Patients were prospectively accrued from September 1, 2005, through January 6, 2022. This prospective follow-up study was approved by the Cleveland Clinic institutional review board. All study participants provided informed written consent to participate. This study followed the Strengthening the Reporting of Observational Studies in Epidemiology (STROBE) reporting guideline for cohort studies. Patients were broadly recruited from community and academic medical centers throughout North America, South America, Europe, Australia, and Asia,^5,9^ and evaluated at the *PTEN* Multidisciplinary Clinic and Center of Excellence at Cleveland Clinic. Inclusion criteria for this study included meeting at minimum the relaxed operational diagnostic criteria of the International Cowden Consortium, meaning missing only 1 feature from the full diagnostic criteria, having macrocephaly plus NDD (ASD, developmental delay, intellectual disability), and positive germline *PTEN* variant testing results.^5,14^ Checklists to document specific clinical features were completed by specialist genetic counselors or physicians, concurrently with withdrawal of blood specimen. Specialist genetics staff reviewed all checklists and if necessary, corresponded with the enrolling center to obtain primary documentation of medical records and pathology reports for phenotypic confirmation with patient consent. Relatives of participants with germline *PTEN* variants were offered genetic counseling and testing.

### Germline *PTEN* Variants and Deletion Analysis

All eligible research participants underwent germline *PTEN* variant and deletion analysis using standard protocols.^9,15^ The *PTEN* variants were classified into tier 1, including all pathogenic and likely pathogenic variants, and tier 2, including variants of uncertain significance (VUS) and those with conflicting interpretation of pathogenicity (at least 1 classification should be pathogenic, likely pathogenic, or a VUS). All benign and likely benign *PTEN* variants were excluded. Pathogenicity estimates were ascertained by clinical genetic testing reports where available, ClinVar database classifications, and/or the ClinGen gene-specific criteria for *PTEN* variant curation.^16^ Analyses were performed with all patients harboring a germline *PTEN* variant, as well as for those with tier 1 and tier 2 *PTEN* variants separately.

### Follow-up Procedures

We followed up this cohort prospectively for the development of phenotypic features associated with PHTS. We recorded baseline information including detailed cancer history at the time of consent. Between July 2021 and July 2022, trained interviewers conducted telephone interviews with individuals with PHTS or their family caregivers to obtain updated phenotypic information in those who had not routinely seen us in genetics clinic within 3 years. We reviewed electronic health records of patients with PHTS internal to the Cleveland Clinic health system. For these cases, the date of the last documented clinical update, regardless of the type of medical visit, was considered the date of last follow-up. Individuals who could not be contacted (eg, due to a change in home address and associated contact information), and those without electronic health record profiles were designated as lost to follow-up and the date of last follow-up annotated as the initial date at consent. Cancer diagnoses, including subsequent SMN, were confirmed by pathology reports whenever possible. There were 14 individuals with missing dates of cancer diagnosis who were excluded from the time to cancer analysis. Cancers in children and young adults were limited to individuals aged 29 years and younger at time of cancer diagnosis.^17,18^ When calculating lifetime cancer risks compared with the general population, individuals having in situ cancers and/or benign tumors were not included in the cancer group which only included invasive cancers beyond stage 0. Ductal breast carcinomas in situ were included in the descriptive characterization of the cancer spectrum in young adults with PHTS and individuals with PHTS and NDD. Additionally, we excluded metastatic disease.^9^ Bilateral breast cancers and unilateral breast cancers of different histology were considered separate primary malignant neoplasms. For the time to second primary cancer analysis, the date of diagnosis of each primary cancer was considered the index or baseline date relative to any subsequent SMN.

### Statistical Analysis

Age at cancer diagnosis was analyzed using a cause-specific approach via the Kaplan-Meier method and differences tested using the log-rank test. Median follow-up was calculated in those participants who did not develop cancer during the observed follow-up period. For this analysis, age at cancer diagnosis was calculated as the number of years from birth to the date of first cancer or date of last follow-up. This analysis was conducted by defining an event as any cancer (overall), as well as by specific cancer types. In a subset analysis of participants who developed a cancer during follow-up, time to second cancer was calculated as years from the date of the first cancer to the date of the second cancer or last follow-up. We calculated standardized incidence ratios (SIR) using age-specific incidence data from the Surveillance Epidemiology and End Results database (2000-2019), and estimated age-related penetrance of cancer using the Kaplan-Meier method.^5^ Analyses for female-specific cancers (breast, endometrial) included only female individuals with PHTS (there was only 1 male participant with breast cancer). All statistical tests were 2-sided, and *P* values <.05 were deemed significant. Analyses were performed using R software version 4.2.1 (R Project for Statistical Computing) and OpenEpi version 3.01 (Open Source Epidemiologic Statistics for Public Health) software. Data were analyzed from July 2022 to February 2023.

## Results

### Patient Characteristics and Germline *PTEN* Variation Spectrum

We prospectively accrued 7302 research participants from September 1, 2005, through January 6, 2022. After undergoing germline *PTEN* variation and deletion analysis, 701 patients were found to have germline *PTEN* variants (median [IQR] age at consent, 38 [12-52] years; 413 female [59%] and 288 male [41%] participants) (Table 1). Longitudinal follow-up data could be obtained for 260 (37%) patients, with a median (IQR) follow-up of 4 (2-8) years. Of the 701 research participants, 341 (49%) received cancer diagnoses, with 144 (42%) of those diagnosed with SMN. There were 29 patients with incident cancers diagnosed after the date of consent (median [IQR] age at diagnosis of a first cancer, 43 [27-51] years). Of the 360 patients without cancer, 190 (53%) received diagnoses of NDD. *PTEN* variants were classified into tier 1 including all pathogenic and likely pathogenic variants (507 variants [72%]), and tier 2 including variants of uncertain significance (VUS) and those with conflicting interpretation of pathogenicity (194 variants [28%]). Missense, nonsense, promoter, and frameshift truncating *PTEN* variants were the most represented in this series (Table 1). Of the 496 exonic coding variants, 291 (59%) were in regions encoding the phosphatase domain, and 174 (35%) were in regions encoding the C2 domain of the PTEN protein.

**Table 1.** Clinical Phenotypic and Genotypic Characteristics of PHTS Patients

Clinical characteristics	Patients, No. (%) (N = 701)
Sex	
Female	413 (59.0)
Male	288 (41.0)
Age at consent, median (IQR)	38 (12-52)
PHTS phenotype classifications	
Cancer	318 (45.4)
NDD^a^	190 (27.1)
Cancer and NDD	23 (3.3)
No cancer and no NDD	170 (24.3)
Germline *PTEN* variant classifications	
Pathogenic and /or likely pathogenic	507 (72.3)
VUS	177 (25.3)
Conflicting pathogenic/VUS	3 (0.43)
Conflicting likely pathogenic/VUS	5 (0.71)
Conflicting VUS/benign	1 (0.14)
Conflicting VUS/likely benign	8 (1.14)
Germline *PTEN* variant spectrum	
Missense	210 (30.0)
Nonsense	135 (19.3)
Promoter	113 (16.1)
Splice site	65 (9.3)
Frameshift truncating	107 (15.3)
Large deletions or duplications	46 (6.6)
Other^b^	25 (3.6)

Abbreviations: NDD, neurodevelopmental disorder; PHTS, *PTEN* hamartoma tumor syndrome; VUS, variant of uncertain significance.

^a^ Includes autism spectrum disorder, developmental delay, and/or intellectual disability.

^b^ Includes inframe deletions (7), inframe insertions (1), indels (4), synonymous variants (2), intronic variants (9), and stop codon loss variants (2).

### Lifetime Cancer Risks

**Figure 1.**
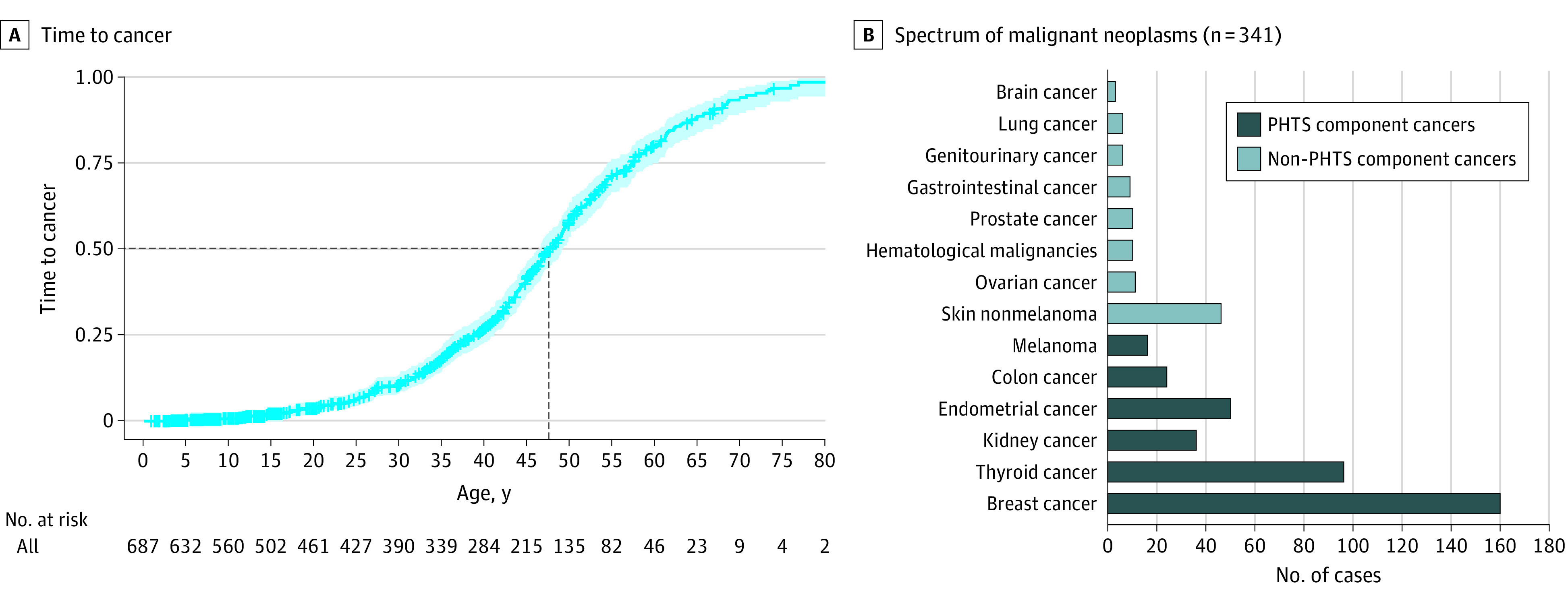
Time to First Cancer and Spectrum of Malignant Neoplasms in Patients With *PTEN* Hamartoma Tumor Syndrome (PHTS) Panel A, Cumulative incidence of the first cancer. Out of 687 participants, 328 developed a cancer. The median age at cancer diagnosis was 47.6 years (95% CI, 46.5-49.3 years). Median (IQR) follow-up for those who did not develop cancer was 15.4 (7.4-35.7) years. Panel B, The types of cancer observed in 341 patients with PHTS.

The analytical series consisted of 687 patients with PHTS after excluding individuals without known cancer diagnosis dates. We performed time to first cancer analysis from the date of birth to identify the cumulative incidence of having any cancer and then of having each of the *PTEN*-related component cancers.^5^ Of the 687 patients, 328 (47.7%) had at least 1 cancer diagnosis. The median age at cancer diagnosis was 47.6 years (95% CI, 46.5-49.3 years) (Figure 1A). The median (IQR) follow-up for those who did not develop cancer was 15.4 (7.4-35.7) years. We observed a wide spectrum of diagnosed cancer types (Figure 1B). Compared with standard population risks, we observed elevated risks of thyroid (age-adjusted SIR 32.1; 95% CI, 26.0-39.0), kidney (SIR 26.5; 95% CI, 18.8-36.3), endometrial (SIR 26.0; 95% CI, 19.5-34.1), breast (SIR 20.3; 95% CI, 17.3-23.7), and colorectal (SIR 7.9; 95% CI, 5.2-11.7) cancers, and melanoma (SIR 6.3; 95% CI, 3.5-10.5). Significantly elevated lifetime cancer risks remain when limiting the analysis to PHTS individuals with tier 1 *PTEN* variants vs the entire patient series including tier 1 and tier 2 *PTEN* variants. Patients with PHTS with tier 2 *PTEN* variants have elevated lifetime cancer risks for all component cancers except for melanoma (Table 2). Age-related penetrance estimates reveal 91% (95% CI, 81%-96%) lifetime risks for invasive female breast cancer, 33% (95% CI, 24%-40%) for thyroid cancer, 48% (95% CI, 21%-65%) for endometrial cancer, 30% (95% CI, 10%-46%) for kidney cancer, 17% (95% CI, 0%-33%) for colorectal cancer, and 5% (95% CI, 2%-7%) for melanoma (Table 3; eFigure 1 in Supplement 1). Of the 341 individuals with PHTS and cancer, 144 (42.2%) developed an SMN at final follow-up. The median (IQR) age at consent was 55 (47-62) years. The median interval between primary cancers and SMN was 13.2 (95% CI, 9.7-18.9) years (eFigure 2 in Supplement 1). There were 94 (65.3%) patients with PHTS who developed more than 2 primary cancers and 43 (29.9%) with more than 3 primary cancers.

**Table 2.** SIRs of Component Cancers in PHTS

Cancer	Expected	Observed	SIR (95% CI)	*P* value
All PHTS with tier 1 and tier 2 *PTEN *variants				
Thyroid	2.93	94	32.1 (26.0-39.0)	<.001
Kidney	1.36	36	26.5 (18.8-36.3)	<.001
Endometrial^a^	1.88	49	26.0 (19.5-34.1)	<.001
Breast^a^	7.69	156	20.3 (17.3-23.7)	<.001
Colorectal	2.90	23	7.9 (5.2-11.7)	<.001
Melanoma	2.06	13	6.3 (3.5-10.5)	<.001
PHTS with tier 1 *PTEN *variants				
Thyroid	1.59	70	44.1 (34.7-55.4)	<.001
Kidney	0.77	30	39.0 (26.8-54.9)	<.001
Endometrial^a^	0.81	37	45.5 (32.5-62.1)	<.001
Breast^a^	3.35	77	23.0 (18.3-28.6)	<.001
Colorectal	1.55	18	11.6 (7.1-18.0)	<.001
Melanoma	1.14	12	10.6 (5.7-18.0)	<.001
PHTS with tier 2 *PTEN* variants				
Thyroid	1.35	24	17.8 (11.7-26.1)	<.001
Kidney	0.59	6	10.2 (4.1-21.1)	<.001
Endometrial^a^	1.07	12	11.2 (6.1-19.0)	<.001
Breast^a^	4.33	79	18.2 (14.5-22.6)	<.001
Colorectal	1.35	5	3.7 (1.4-8.2)	.02
Melanoma	0.92	1	1.1 (.05-5.3)	.84

Abbreviations: PHTS, *PTEN *hamartoma tumor syndrome; SIR, standardized incidence rate.

^a^ Includes only female participants.

**Table 3.** Lifetime Cancer Risks of Component Cancers in PHTS

Cancer	Lifetime risk (95% CI), %^a^	Median age at diagnosis (range: lowest to highest age), y^b^
All PHTS with tier 1 and tier 2 *PTEN* variants		
Thyroid	33 (24-40)	37 (7-69)
Kidney	30 (10-46)	52 (21-76)
Endometrial^c^	48 (21-65)	49 (21-78)
Breast^c^	91 (81-96)	49 (16-85)
Colorectal	17 (0-33)	45 (21-77)
Melanoma	5 (2-7)	41 (20-84)
PHTS with tier 1 *PTEN* variants		
Thyroid	40 (24-52)	36 (7-69)
Kidney	31 (18-42)	50 (21-64)
Endometrial^c^	50 (24-67)	45 (21-69)
Breast^c^	89 (73-96)	48 (16-75)
Colorectal	12 (6-18)	46 (21-59)
Melanoma	7 (2-12)	36 (20-60)
PHTS with tier 2 *PTEN* variants		
Thyroid	24 (13-33)	42 (10-63)
Kidney	23 (0-45)	58 (46-76)
Endometrial^c^	40 (3-63)	59 (41-78)
Breast^c^	88 (73-95)	50 (27-85)
Colorectal	17 (0-39)	44 (43-77)
Melanoma	1 (0-3)	65 (45-84)

Abbreviation: PHTS, *PTEN *hamartoma tumor syndrome.

^a^ Lifetime cancer risks represent cumulative incidence rates by age 80 years, except for breast cancer risk with tier 1 *PTEN* variants which represents cumulative incidence rates by age 75 years.

^b^ Median (range: most frequently diagnosed age ranges) in years from the Surveillance Epidemiology and End Results database (2015-2019, all races, both sexes; accessed on February 16, 2023): thyroid cancer 51 (45-54) years, kidney cancer 65 (65-74) years, endometrial cancer 63 (55-64) years, breast cancer 63 (65-74) years, colorectal cancer 66 (65-74) years, melanoma of the skin 65 (65-74) years.

^c^ Includes only female participants.

Higher lifetime risks of developing any cancer were found to be associated with tier 1 *PTEN* variants, including truncating *PTEN* variants, whereas lower lifetime risks of any cancer were associated with *PTEN* promoter variants (eFigure 3 in Supplement 1). Of all 6 PHTS component cancers, the lifetime risk of breast cancer was not different between individuals with tier 1 *PTEN* variants vs those with tier 2 *PTEN* variants. However, those with tier 2 *PTEN* variants had lower lifetime risks of kidney, endometrial, thyroid, and colorectal cancers, as well as melanoma compared with those with tier 1 *PTEN* variants (eFigure 4 in Supplement 1). Additionally, the presence of truncating *PTEN* variants was associated with higher lifetime risks of colorectal, thyroid, breast, and endometrial cancers (eFigure 5 in Supplement 1). The presence of *PTEN* promoter variants was associated with lower lifetime risks of kidney, endometrial, breast, and thyroid cancers (eFigure 6 in Supplement 1).

### Cancer Spectrum in Children, Young Adults, and Individuals With Neurodevelopmental Disorders

**Figure 2.**
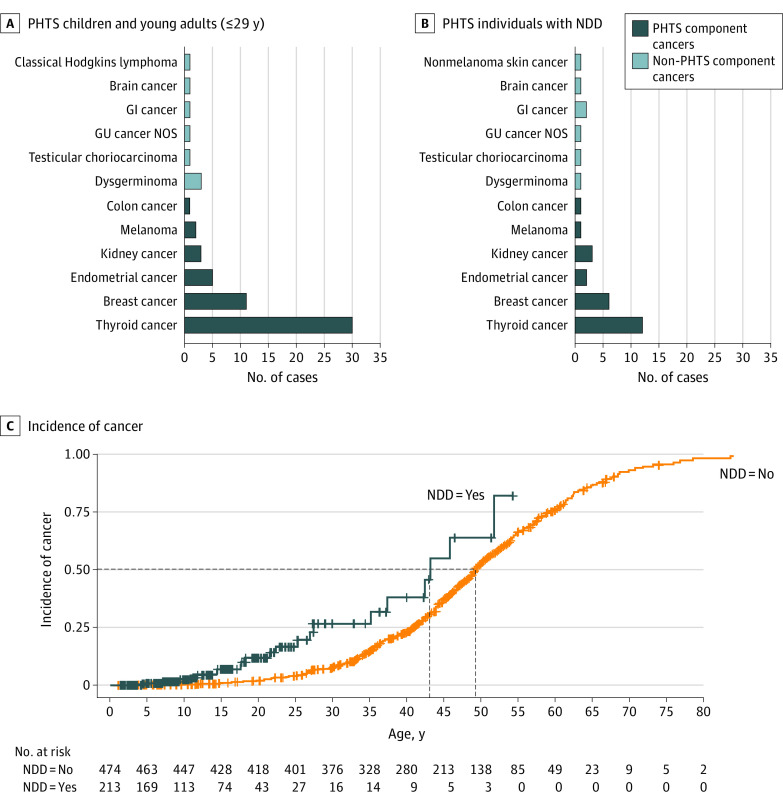
Cancer Spectrum in Children, Young Adults, and Patients With *PTEN* Hamartoma Tumor Syndrome (PHTS) and Neurodevelopmental Disorders (NDD) Panel A, Types of cancer observed in 51 patients with PHTS who had at least 1 cancer diagnosis at or younger than 29 years. Panel B, Types of cancer observed in 23 patients with PHTS who had both cancer and neurodevelopmental disorders. Panel C, Kaplan-Meier curve showing the incidence of any cancer in patients with PHTS with neurodevelopmental disorders compared with those without neurodevelopmental disorders. GI indicates gastrointestinal; GU, genitourinary; NOS, not otherwise specified.

Of the 341 patients with PHTS with cancer, 51 (15%) had at least 1 cancer diagnosis at age 29 years or younger. This group of young patients with cancer consisted of 42 (82.4%) female and 9 (17.6%) male patients. The median (IQR) age at consent for this group was 28 (22-38) years, while the median (IQR) age at cancer diagnosis was 24 (17-26) years. The most prevalent cancer observed in 59% of these patients was thyroid cancer (Figure 2A). Tier 1 *PTEN* variants were observed in 42 (82.4%) of these patients. Of the 51 patients with early-onset cancer, 16 (31.4%) developed SMN at final follow-up, with a median (IQR) time to subsequent cancer diagnosis of 16 (5-22) years.

Regardless of age at cancer diagnosis, we identified 23 of 213 patients with NDD who had both NDD and cancer (11%). This group consisted of 15 (65%) female and 8 (35%) male patients. The median (IQR) age at consent for this group was 29 (21-45) years, with 16 (70%) of the patients presenting with the first cancer at or before age 29 years. The median (IQR) age at cancer diagnosis was 25 (16-43) years. Tier 1 *PTEN* variants were observed in 22 patients (96%). Thyroid cancer was the most prevalent cancer observed in 52% of these patients with PHTS with NDD and cancer (Figure 2B). Of these 23 patients with NDD and cancer, 5 (22%) developed SMN at final follow-up, with a median (IQR) time to subsequent cancer diagnosis of 4 (1-14) years. Individuals with PHTS and NDD showed higher lifetime cancer risks compared with individuals with PHTS but without NDD (hazard ratio, 2.7; 95% CI, 1.7-4.2; *P* < .001) (Figure 2C). This observation held true after accounting for biological sex (eTable in Supplement 1).

## Discussion

In this study, we report cancer risk estimates from the longitudinal follow-up of an international prospective series of pediatric and adult patients with PHTS. Even before the mapping and discovery of *PTEN* as the first susceptibility gene for Cowden syndrome, it was recognized that patients with Cowden syndrome have elevated lifetime risks of developing breast and thyroid cancers.^19,20^ Subsequent systematic studies showed an association with increased lifetime risks of 4 additional cancers, including endometrial cancer, kidney cancer, colorectal cancer, and melanoma.^5,6,7,8^ These studies provided evidence-based surveillance strategies that form the basis of national practice guidelines.^21^ Since 2000, the International Cowden Consortium has been prospectively accruing patients from international centers for the purposes of studying the natural history of PHTS.^1,22^ Compared with our initial analysis of cancer risks in PHTS,^5^ this study includes an expanded series of patients, has a longitudinal follow-up component on more than one third of research participants, and follows standard *PTEN* classification criteria that were previously nonexistent.^16^

Following the prospective accrual of a larger number of patients over more than 20 years for this study, we show a consistent association with increased lifetime risks of developing any cancer, and of developing the known PHTS component cancers, including female breast cancer (91%), thyroid cancer (33%), endometrial cancer (48%), kidney cancer (30%), colorectal cancer (17%), as well as melanoma (5%). Higher lifetime risks of any cancer were associated with truncating *PTEN* variants and with all tier 1 variants together. Importantly, individuals with tier 1 and tier 2 *PTEN* variants had increased lifetime cancer risks compared with the general population (tier 1 > tier 2 > general population). Moreover, whereas we did not observe a difference in lifetime breast cancer risk between patients with tier 1 vs tier 2 *PTEN* variants, those with tier 2 *PTEN* variants had lower lifetime risks of all other component cancer types, although the risks of thyroid, breast, endometrial, and kidney cancers remain significantly higher than risks in the general population. Relatedly, truncating and promoter *PTEN* variants also showed a pattern of different lifetime cancer risk estimates according to cancer type. Specifically, the lifetime risks for colorectal cancer, thyroid cancer, breast cancer, and endometrial cancer were higher in individuals with truncating *PTEN* variants compared with those without truncating *PTEN* variants. Conversely, the presence of *PTEN* promoter variants was associated with lower lifetime risks of kidney, endometrial, breast, and thyroid cancers compared with those without *PTEN* promoter variants. We did not identify robust differences in lifetime cancer risks according to whether the *PTEN* variants affected the phosphatase domain or the C2 domain of the protein.

Importantly, another knowledge gap in the *PTEN* community is whether individuals with PHTS and NDD have similar lifetime cancer risks compared with neurotypical individuals with PHTS. This is a challenging question to address because the majority of individuals with PHTS and NDD in our study are too young to develop adult onset cancers. Despite the limited sample size, we found that patients with PHTS with NDD have a higher lifetime risk of cancer compared with patients with PHTS without NDD. However, it will be important to validate this finding. Additionally, we present descriptive data on the clinical characteristics and spectrum of cancers encountered in 51 patients with PHTS having at least 1 cancer diagnosed at or younger than 29 years, and 23 patients with PHTS having both NDD and cancer (16 patients overlap between both groups). Importantly, we found that approximately 20% to 30% of these patients have already developed SMN at final follow-up. Longitudinal studies to characterize cancer risks in younger individuals with PHTS and in those with NDD are warranted. This is an ongoing clinical trial in which pediatric and adult patients with PHTS with or without NDD are followed throughout their lifespan for other manifestations with age-related penetrance, such as cancer (Natural History Study of Individuals with Autism and Germline Heterozygous *PTEN* Mutations^23^).

### Limitations

This study had limitations. Ascertainment bias is a possible limitation when evaluating patients with rare syndromes such as PHTS who seek care at a Center of Excellence. We have sought to minimize this through inclusion of asymptomatic family members with germline *PTEN* variants, individuals with PHTS who have had prophylactic surgerical procedures, and through the nature of referrals to the *PTEN* Multidisciplinary Clinic, which are representative of medical genetics vs an oncology practice, with the latter biasing for cancer diagnoses.

## Conclusions

According to the presented data, we recommend the continuation of enhanced organ-specific cancer surveillance, including in patients with tier 2 germline *PTEN* variants.^21^ The consistently elevated lifetime cancer risks compared with more recent studies in the European population^8^ highlight the need for multiancestry studies that inform standard universal guidelines for PHTS management. The latter is a forthcoming international effort to be guided by this and other studies on cancer risks in individuals with PHTS.

## Notes

Supplement 1. **eFigure 1. **Lifetime Cancer Risks in Patients with *PTEN* Hamartoma Tumor Syndrome **eFigure 2. **Time for the Development of Second Primary Malignant Neoplasms in Patients With *PTEN* Hamartoma Tumor Syndrome **eFigure 3. **Lifetime Risks of All Cancers According to *PTEN *Variant Status **eFigure 4. **Lifetime Risks of PHTS Component Cancers According to *PTEN *Variant Tier **eFigure 5. **Elevated Lifetime Risks of Organ-Specific Cancers in Patients With Truncating *PTEN *Variants **eFigure 6. **Lower Lifetime Risks of Organ-Specific Cancers in Patients With *PTEN *Promoter Variants **eTable. **Regression Models of Cancer Risks by NDD Status After Accounting for Biological Sex

Supplement 2.
Data Sharing Statement
