# Supplementary material for: Longitudinal Analysis of Cancer Risk in Children and Adults With Germline PTEN Variants
Source: JAMA Netw Open. 2023 Apr 24;6(4):e239705. doi: 10.1001/jamanetworkopen.2023.9705 (PMC10126871; doi:10.1001/jamanetworkopen.2023.9705)
Supplement: Supplement 1. — eFigure 1. Lifetime Cancer Risks in Patients with PTEN Hamartoma Tumor Syndrome eFigure 2. Time for the Development of Second Primary Malignant Neoplasms in Patients With PTEN Hamartoma Tumor Syndrome eFigure 3. Lifetime Risks of All Cancers According to PTEN Variant Status eFigure 4. Lifetime Risks of PHTS Component Cancers According to PTEN Variant Tier eFigure 5. Elevated Lifetime Risks of Organ-Specific Cancers in Patients With Truncating PTEN Variants eFigure 6. Lower Lifetime Risks of Organ-Specific Cancers in Patients With PTEN Promoter Variants eTable. Regression Models of Cancer Risks by NDD Status After Accounting for Biological Sex [file jamanetwopen-e239705-s001.pdf]

Supplemental Online Content

Yehia L, Plitt G, Tushar AM, et al. Longitudinal analysis of cancer risk in children and adults with germline *PTEN* variants. *JAMA Netw Open*. 2023;6(4):e239705. doi:10.1001/jamanetworkopen.2023.9705

eFigure 1. Lifetime Cancer Risks in Patients with *PTEN* Hamartoma Tumor Syndrome

eFigure 2. Time for the Development of Second Primary Malignant Neoplasms in Patients With *PTEN* Hamartoma Tumor Syndrome

eFigure 3. Lifetime Risks of All Cancers According to *PTEN* Variant Status

eFigure 4. Lifetime Risks of PHTS Component Cancers According to *PTEN* Variant Tier

eFigure 5. Elevated Lifetime Risks of Organ-Specific Cancers in Patients With Truncating *PTEN* Variants

eFigure 6. Lower Lifetime Risks of Organ-Specific Cancers in Patients With *PTEN* Promoter Variants

eTable. Regression Models of Cancer Risks by NDD Status After Accounting for Biological Sex

This supplemental material has been provided by the authors to give readers additional information about their work.

© 2023 Yehia L et al. *JAMA Network Open.*

eFigure 1. Lifetime Cancer Risks in Patients with *PTEN* Hamartoma Tumor Syndrome

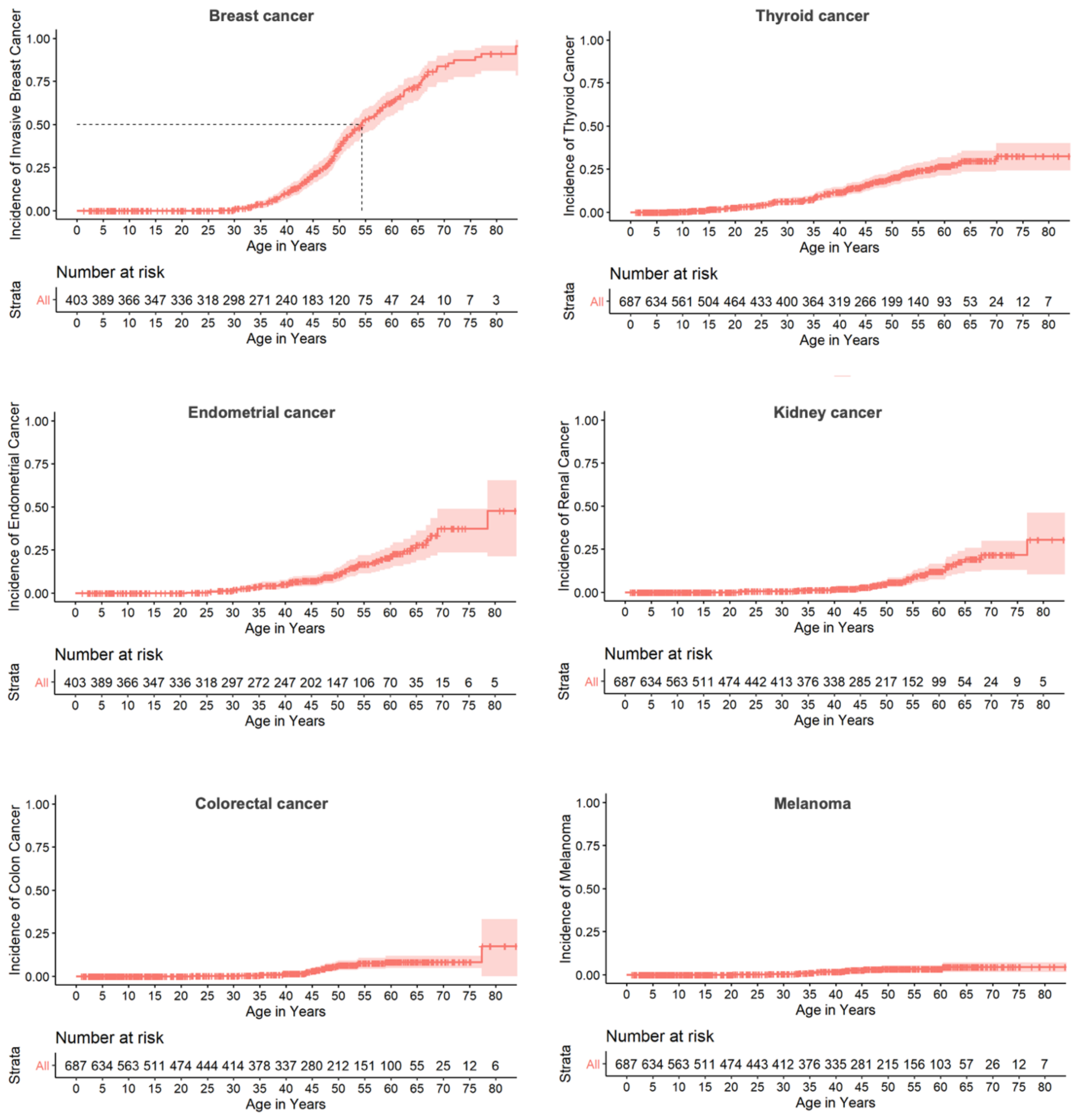

Age-related penetrance curves for female breast cancer, thyroid cancer, endometrial cancer, kidney cancer, colorectal cancer, and melanoma. The highest age-related penetrance is observed in female breast cancer, with an estimated 91% lifetime risk.

© 2023 Yehia L et al. *JAMA Network Open.*

eFigure 2. Time for the Development of Second Primary Malignant Neoplasms in Patients With *PTEN* Hamartoma Tumor Syndrome

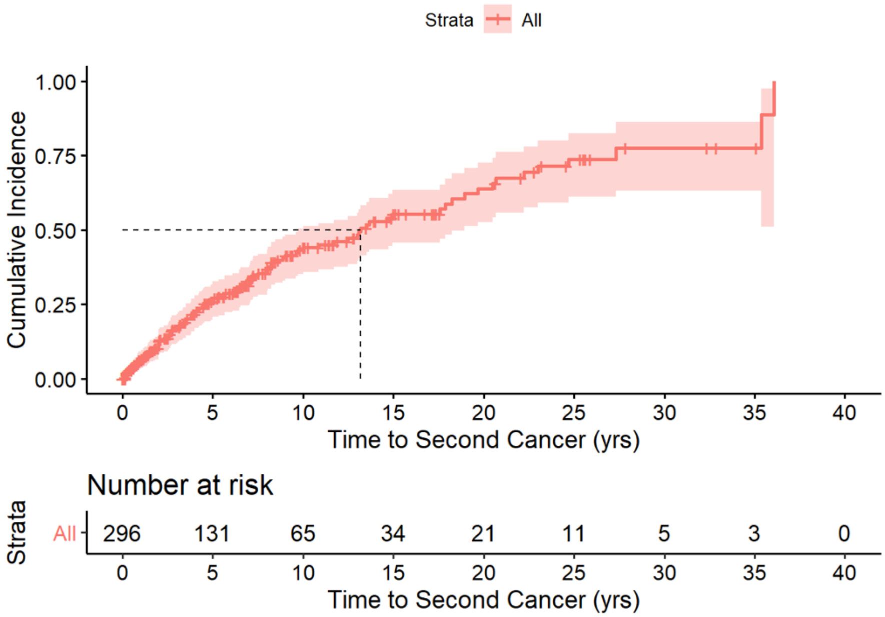

Of the 341 individuals with PHTS and cancer, 144 developed at least one more malignant neoplasm at final follow-up. The median interval between primary cancers and second cancers was 13.2 years (95% CI 9.7 - 18.9 years). The x-axis represents the time from first cancers to second cancers in years.

© 2023 Yehia L et al. *JAMA Network Open.*

eFigure 3. Lifetime Risks of All Cancers According to *PTEN* Variant Status

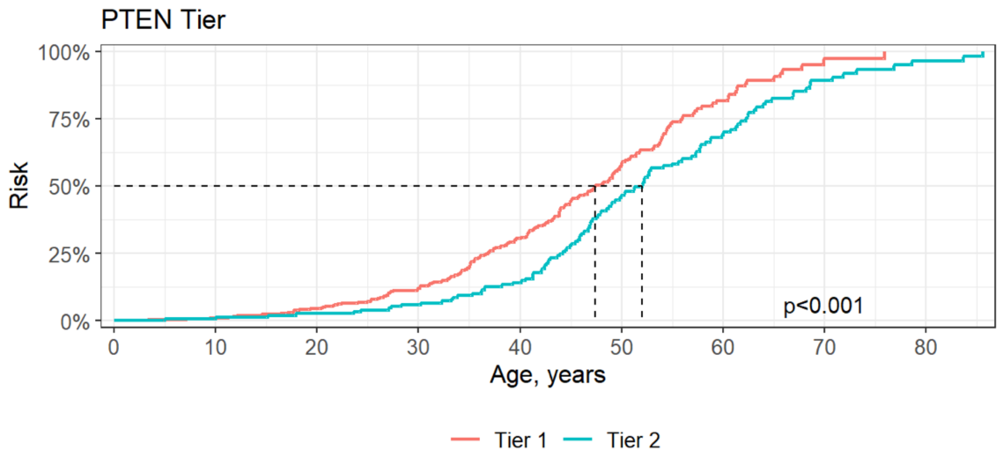

Tier 1									
At Risk	507	404	313	251	168	75	18	1	0
Events	0	4	16	39	89	148	180	192	193

Tier 2									
At Risk	180	156	148	141	121	66	31	8	2
Events	0	1	4	9	21	64	90	108	113

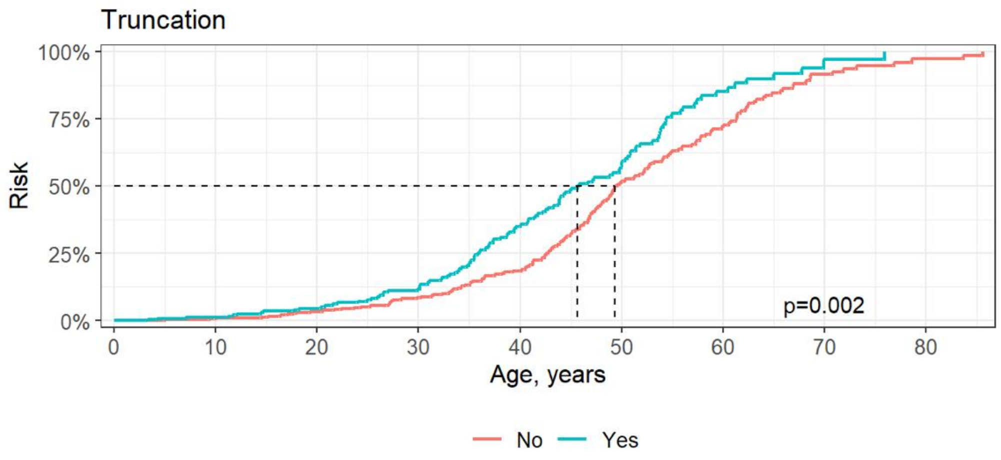

No									
At Risk	394	320	275	243	191	94	39	8	2
Events	0	2	10	24	49	119	154	178	183

Yes									
At Risk	283	232	180	145	95	45	10	1	0
Events	0	3	10	23	60	91	113	119	120

© 2023 Yehia L et al. *JAMA Network Open.*

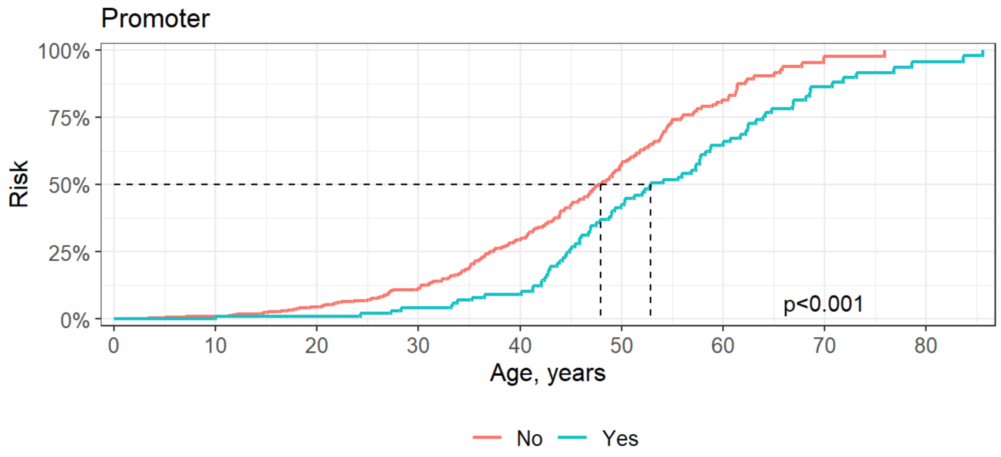

**No**									
At Risk	578	458	361	296	202	90	22	1	0
Events	0	5	19	44	101	172	211	226	227
**Yes**									
At Risk	109	102	100	96	87	51	27	8	2
Events	0	0	1	4	9	40	59	74	79

Higher lifetime risks of developing any cancer were found to be associated with Tier 1 *PTEN* variants (*P*<0.001), including truncating *PTEN* variants (*P*=0.002), whereas lower lifetime risks of any cancer were associated with *PTEN* promoter variants (*P*<0.001).

© 2023 Yehia L et al. *JAMA Network Open.*

eFigure 4: Lifetime Risks of PHTS Component Cancers According to *PTEN* Variant Tier

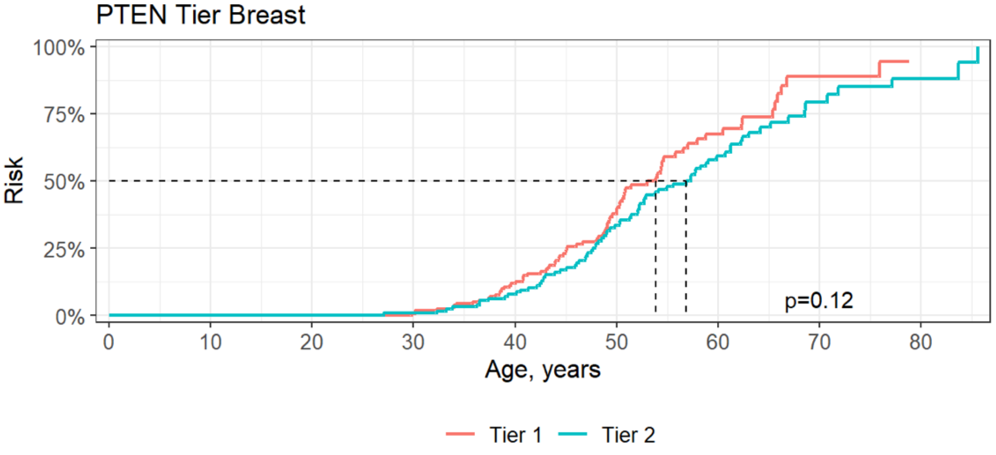

Tier 1

At Risk	261	232	203	167	126	51	17	2	0
Events	0	0	0	1	19	49	68	76	77

Tier 2

At Risk	142	134	133	131	114	69	30	8	3
Events	0	0	0	1	10	39	63	74	77

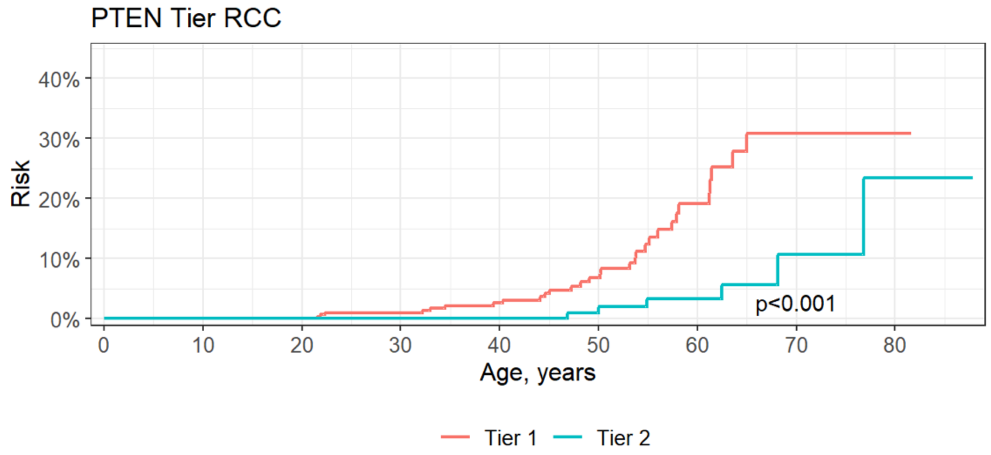

Tier 1

At Risk	507	407	324	267	209	124	45	9	1
Events	0	0	0	3	7	14	25	30	30

Tier 2

At Risk	180	156	150	146	129	93	54	15	4
Events	0	0	0	0	0	1	3	5	6

© 2023 Yehia L et al. *JAMA Network Open.*

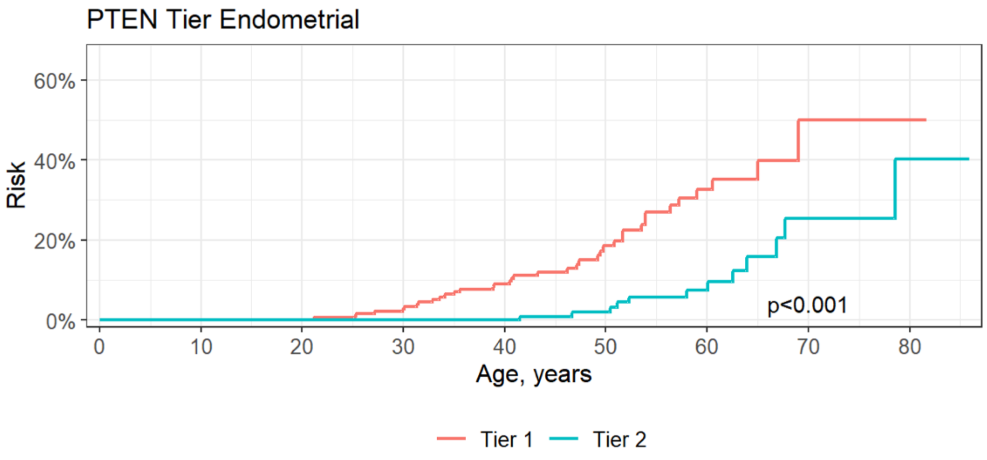

Tier 1

At Risk	261	232	203	166	130	65	27	4	1
Events	0	0	0	5	15	25	34	37	37

Tier 2

At Risk	142	134	133	131	117	82	43	11	4
Events	0	0	0	0	0	2	6	11	12

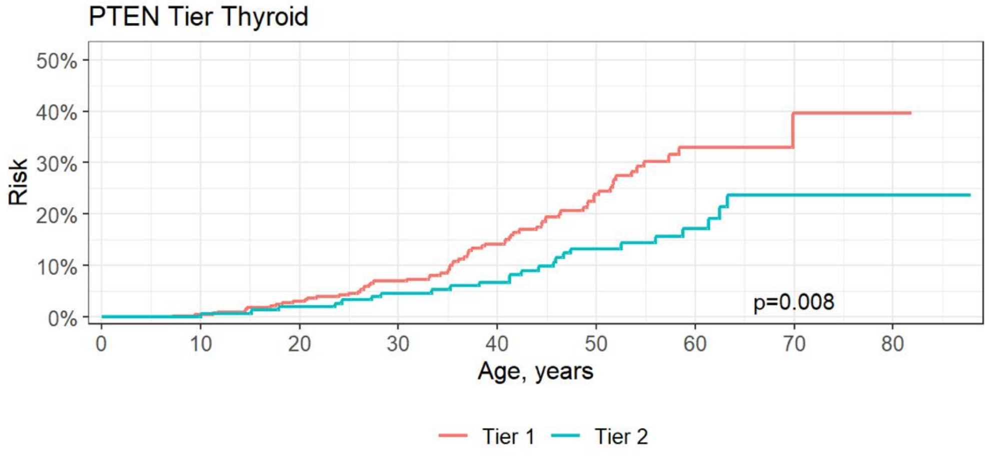

Tier 1

At Risk	507	405	316	259	193	113	44	9	2
Events	0	2	11	23	41	59	69	70	70

Tier 2

At Risk	180	156	148	141	126	86	49	15	5
Events	0	0	3	7	10	18	21	24	24

© 2023 Yehia L et al. *JAMA Network Open.*

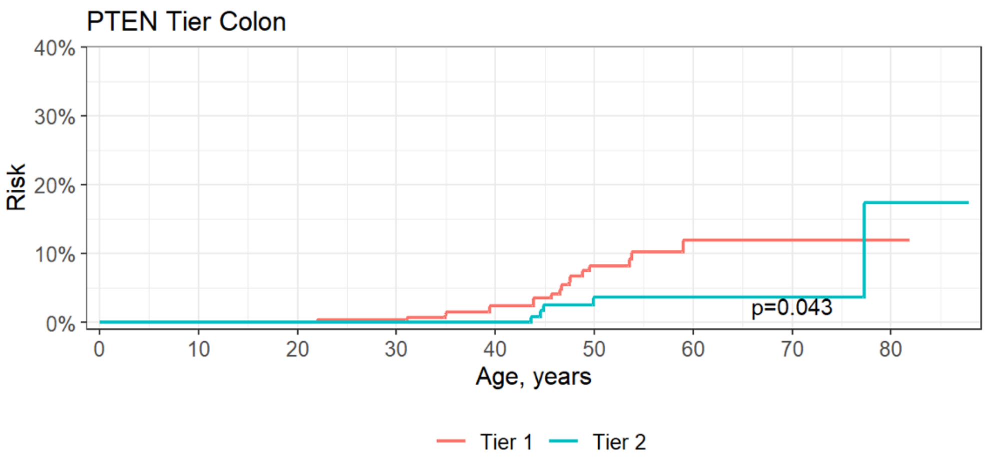

Tier 1

At Risk	507	407	324	268	208	120	47	9	2
Events	0	0	0	1	6	15	18	18	18

Tier 2

At Risk	180	156	150	146	129	92	53	16	4
Events	0	0	0	0	0	4	4	4	5

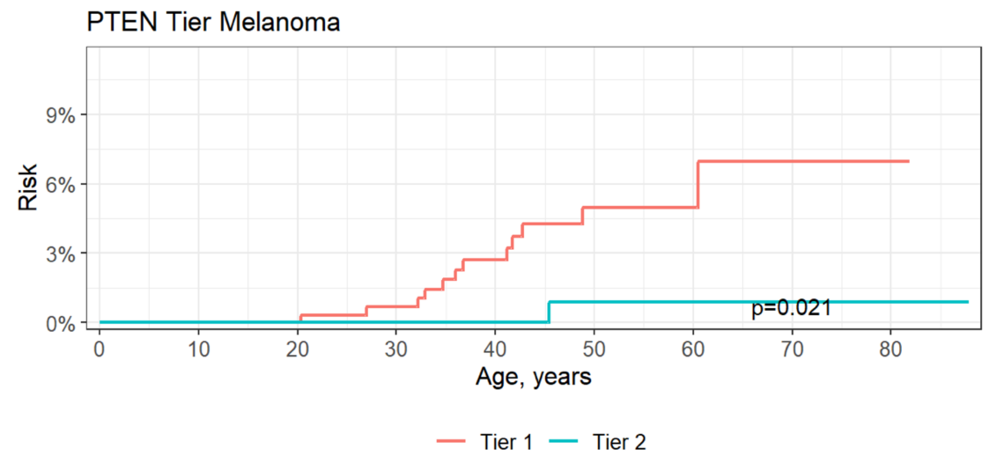

Tier 1

At Risk	507	407	324	266	206	122	50	11	2
Events	0	0	0	2	7	11	11	12	12

Tier 2

At Risk	180	156	150	146	129	93	53	15	5
Events	0	0	0	0	0	1	1	1	1

Red curves depict patients with Tier 1 *PTEN* variants, while the blue curves depict patients with Tier 2 *PTEN* variants.

© 2023 Yehia L et al. *JAMA Network Open.*

eFigure 5. Elevated Lifetime Risks of Organ-Specific Cancers in Patients With Truncating *PTEN* Variants

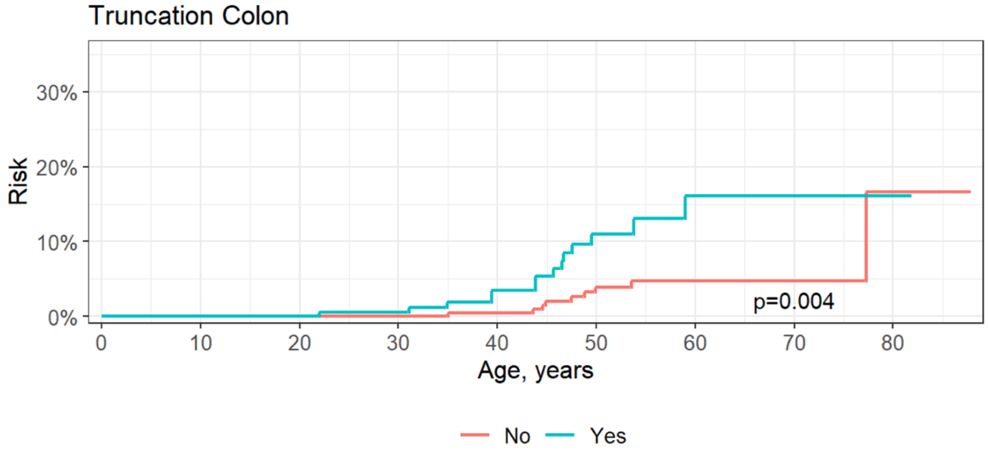

No

At Risk	394	321	281	257	213	145	73	20	4
Events	0	0	0	0	1	7	8	8	9

Yes

At Risk	283	234	187	153	121	65	26	5	2
Events	0	0	0	1	5	12	14	14	14

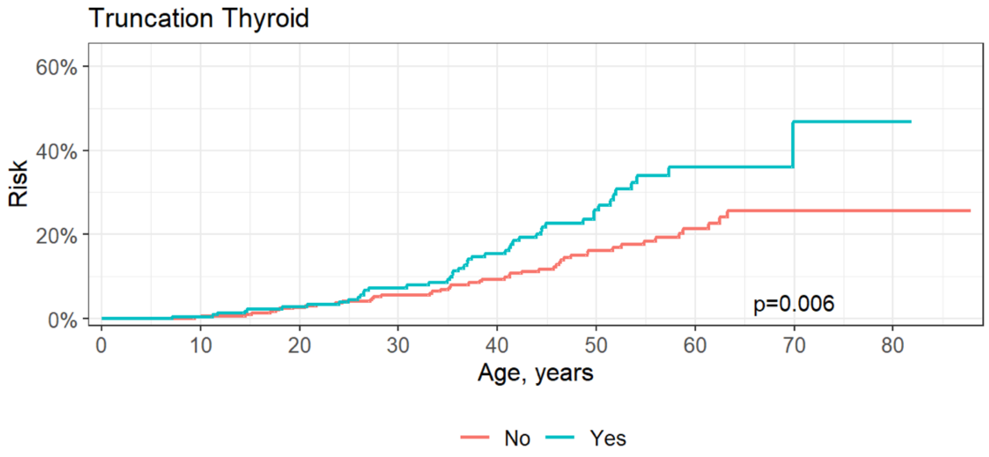

No

At Risk	394	320	276	247	202	132	66	19	5
Events	0	1	8	16	25	38	44	47	47

Yes

At Risk	283	233	182	149	114	65	26	5	2
Events	0	1	6	14	26	38	45	46	46

© 2023 Yehia L et al. *JAMA Network Open.*

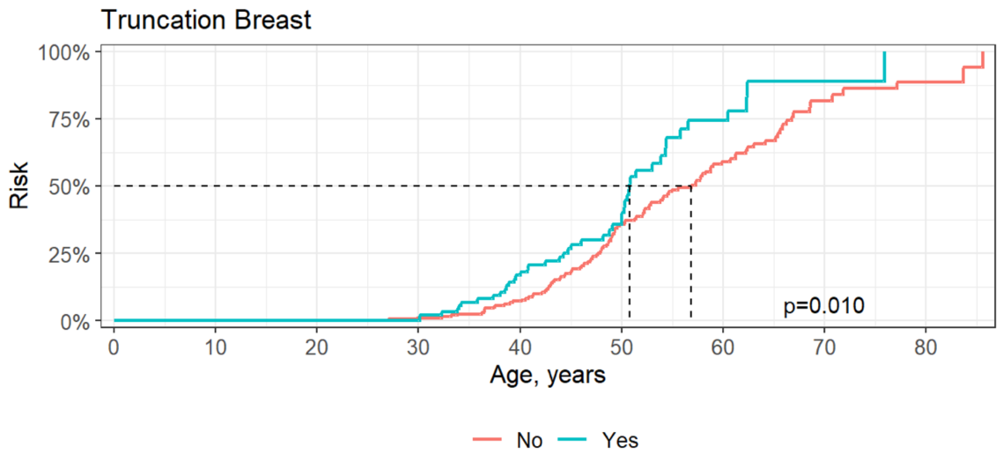

No									
At Risk	252	231	218	204	172	92	40	9	3
Events	0	0	0	2	14	60	89	105	108
Yes									
At Risk	147	131	115	92	66	27	7	1	0
Events	0	0	0	0	15	28	41	44	45

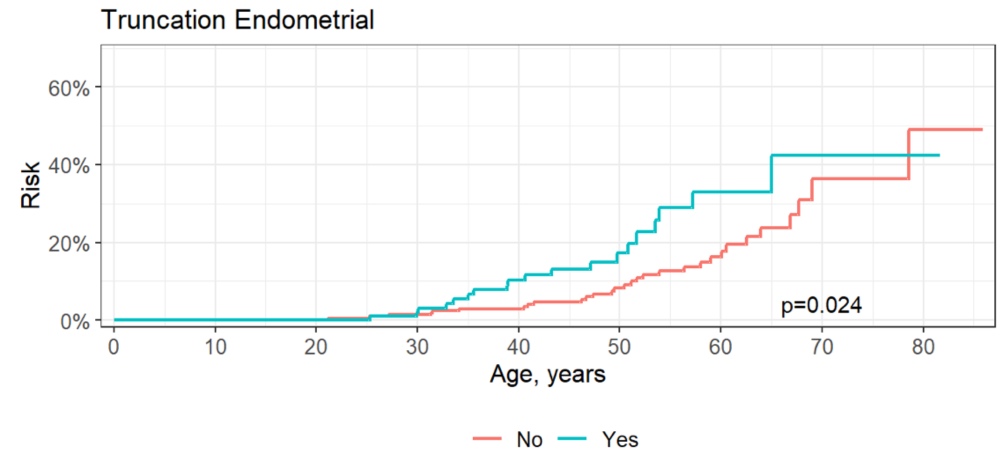

No									
At Risk	252	231	218	204	176	112	54	12	4
Events	0	0	0	3	6	14	22	29	30
Yes									
At Risk	147	131	115	91	69	34	15	3	1
Events	0	0	0	2	9	13	18	19	19

Red curves depict patients without truncating *PTEN* variants, while the blue curves depict patients with truncating *PTEN* variants.

© 2023 Yehia L et al. *JAMA Network Open.*

eFigure 6. Lower Lifetime Risks of Organ-Specific Cancers in Patients With *PTEN* Promoter Variants

Promoter RCC

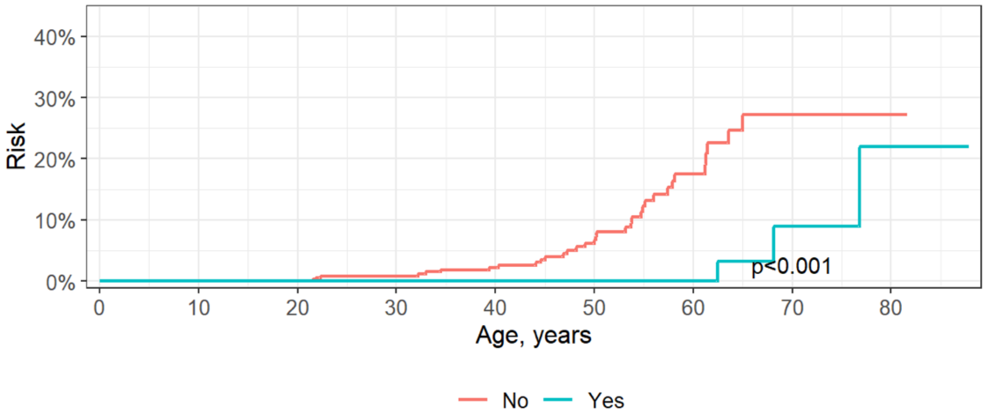

No									
At Risk	578	461	374	315	251	153	58	11	1
Events	0	0	0	3	7	15	28	33	33
Yes									
At Risk	109	102	100	98	87	64	41	13	4
Events	0	0	0	0	0	0	0	2	3

Promoter Endometrial

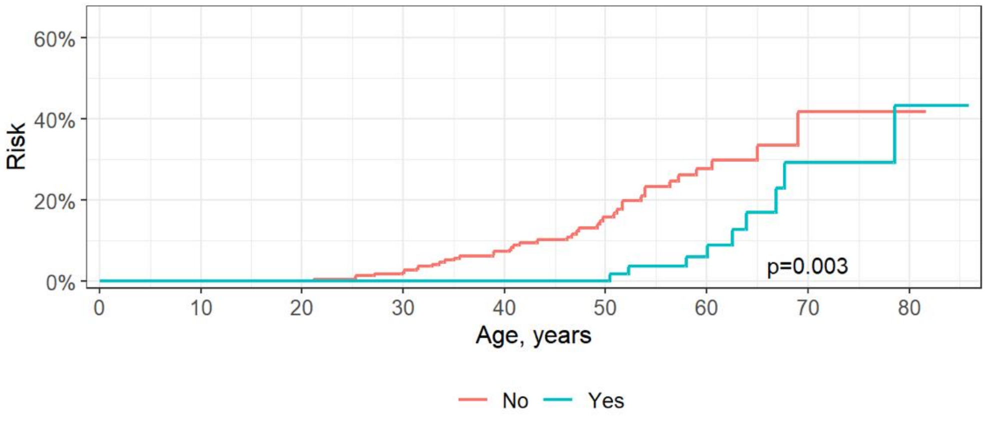

No									
At Risk	309	275	246	208	168	90	38	6	1
Events	0	0	0	5	15	27	37	40	40
Yes									
At Risk	94	91	90	89	79	57	32	9	4
Events	0	0	0	0	0	0	3	8	9

© 2023 Yehia L et al. *JAMA Network Open.*

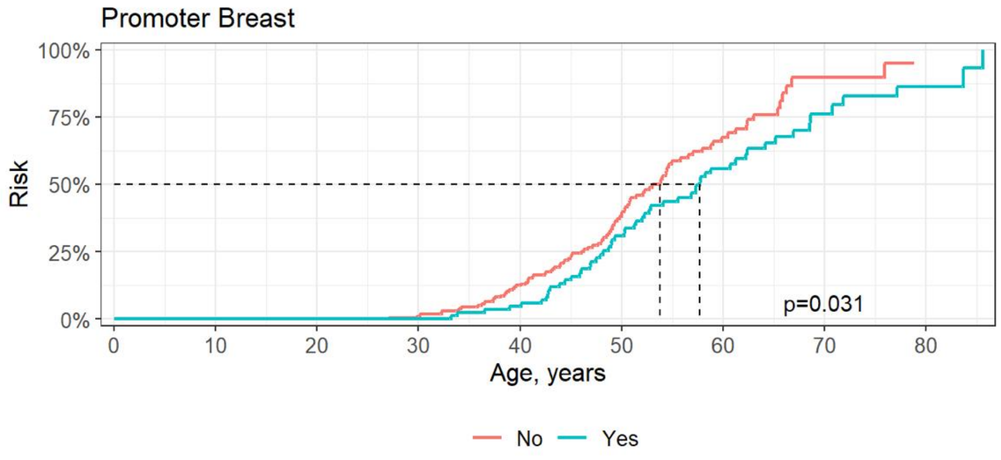

No									
At Risk	309	275	246	209	161	70	22	2	0
Events	0	0	0	2	25	64	90	100	101
Yes									
At Risk	94	91	90	89	79	50	25	8	3
Events	0	0	0	0	4	24	41	50	53

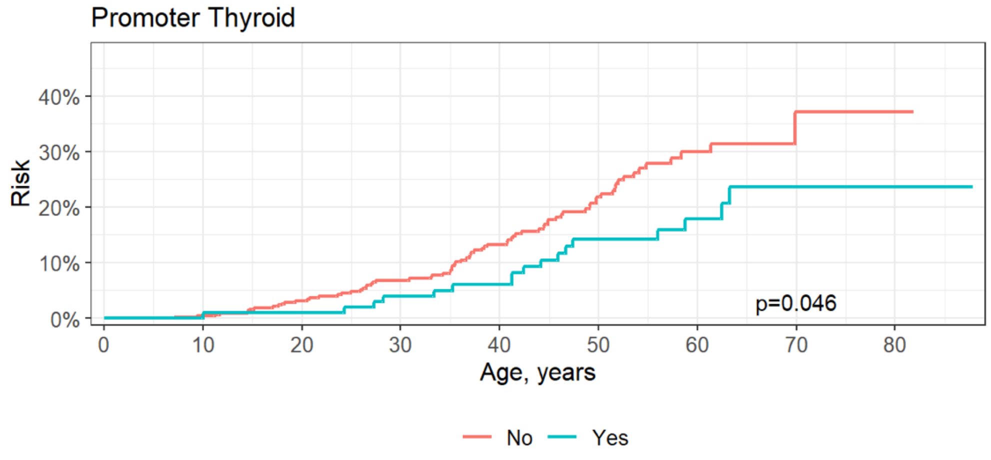

No									
At Risk	578	459	364	304	232	140	55	11	2
Events	0	2	13	26	45	64	75	77	77
Yes									
At Risk	109	102	100	96	87	59	38	13	5
Events	0	0	1	4	6	13	15	17	17

Red curves depict patients without *PTEN* promoter variants, while the blue curves depict patients with *PTEN* promoter variants.

© 2023 Yehia L et al. *JAMA Network Open.*

eTable. Regression Models of Cancer Risks by NDD Status After Accounting for Biological Sex

			Univariable			Multivariable		
**Characteristic**	**N**	**Event N**	**HR**[1]	**95% CI**[1]	**p-value**	**HR**[1]	**95% CI**[1]	**p-value**
NDD	686	307						<0.001
No			—	—		—	—	
Yes			2.34	1.48, 3.70	<0.001	2.68	1.70, 4.24	
Sex	686	307						<0.001
Female			—	—		—	—	
Male			0.57	0.43, 0.76	<0.001	0.54	0.40, 0.72	

[1] HR = Hazard Ratio, CI = Confidence Interval

© 2023 Yehia L et al. *JAMA Network Open.*
