# Supplementary material for: Longitudinal Analysis of Cancer Risk in Children and Adults With Germline PTEN Variants
Source: JAMA Netw Open. 2023 Apr 24;6(4):e239705. doi: 10.1001/jamanetworkopen.2023.9705 (PMC10126871; doi:10.1001/jamanetworkopen.2023.9705)
Supplement: Supplement 2. — Data Sharing Statement [file jamanetwopen-e239705-s002.pdf]

Data Sharing Statement

Yehia. Longitudinal Analysis of Cancer Risk in Children and Adults With Germline PTEN Variants. *JAMA Netw Open*. Published April 24, 2023.
doi:10.1001/jamanetworkopen.2023.9705

Data
Data available: Yes
Data types: Deidentified participant data
How to access data: PTEN genotype Cancer risk data
When available: With publication

Supporting Documents
Document types: None

Additional Information
Who can access the data: N/A
Types of analyses: None
Mechanisms of data availability: With investigator support and signed data access agreement
